# Role of patient and public involvement in implementation research: a consensus study

**DOI:** 10.1136/bmjqs-2017-006954

**Published:** 2018-04-17

**Authors:** Kara A Gray-Burrows, Thomas A Willis, Robbie Foy, Martin Rathfelder, Pauline Bland, Allison Chin, Susan Hodgson, Gus Ibegbuna, Graham Prestwich, Kirsty Samuel, Laurence Wood, Farhat Yaqoob, Rosemary R C McEachan

**Affiliations:** 1 School of Dentistry, University of Leeds, Leeds, UK; 2 Leeds Institute of Health Sciences, University of Leeds, Leeds, UK; 3 ASPIRE PPI Panel, Leeds Institute for Health Sciences, University of Leeds, Leeds, UK; 4 Bradford Institute for Health Research, Bradford Teaching Hospitals NHS Foundation Trust, BD9 6RJ., Bradford, UK

**Keywords:** Implementation research, clinical research, patient and public involvement (PPI), health professionals, consensus panel

## Abstract

**Background:**

Patient and public involvement (PPI) is often an essential requirement for research funding. Distinctions can be drawn between clinical research, which generally focuses on patients, and implementation research, which generally focuses on health professional behaviour. There is uncertainty about the role of PPI in this latter field. We explored and defined the roles of PPI in implementation research to inform relevant good practice guidance.

**Methods:**

We used a structured consensus process using a convenience sample panel of nine experienced PPI and two researcher members. We drew on available literature to identify 21 PPI research roles. The panel rated their agreement with roles independently online in relation to both implementation and clinical research. Disagreements were discussed at a face-to-face meeting prior to a second online rating of all roles. Median scores were calculated and a final meeting held to review findings and consider recommendations.

**Results:**

Ten panellists completed the consensus process. For clinical research, there was strong support and consensus for the role of PPI throughout most of the research process. For implementation research, there were eight roles with consensus and strong support, seven roles with consensus but weaker support and six roles with no consensus. There were more disagreements relating to PPI roles in implementation research compared with clinical research. PPI was rated as contributing less to the design and management of implementation research than for clinical research.

**Conclusions:**

The roles of PPI need to be tailored according to the nature of research to ensure authentic and appropriate involvement. We provide a framework to guide the planning, conduct and reporting of PPI in implementation research, and encourage further research to evaluate its use.

## Introduction

Patient and public involvement (PPI) in research is recognised as best practice and is now an essential requirement to receive funding from many funders globally, including the UK, the Netherlands, Canada, Australia and the USA.^1^ Indeed, the UK National Institute for Health Research (NIHR) supports the INVOLVE programme,^2^ established in 1996 to support active public involvement in healthcare, public health and social care research. INVOLVE aims to advance the role of PPI in all aspects of the research process, including research prioritisation, design, conduct and dissemination. INVOLVE defines involvement as research being carried out ‘with’ or ‘by’ members of the public, rather than ‘to’, ‘about’ or ‘for’ them; the term ‘public’ includes patients, potential patients, carers and people who use health and social care services, as well as organisations that represent people who use these services, but excluded from this definition are people who have a professional role in health and social care services.^3^ The main argument for PPI is that it produces better quality research through influences on, for example, the identification of appropriate research priorities, study marketing and research design (eg, 3–5). This ensures that research is relevant to user needs and hence more likely to have beneficial impacts. There is a further case that the public has a right to be involved in the conduct, management and governance of publicly funded research.^6^ However, there has been little systematic evaluation of PPI and therefore evidence of the impact of PPI on research is limited.^4 7 8^ Further, published reviews on the scope and impact of PPI on research have been limited to clinical-based research,^4 7 8^ and the potential role of PPI in different types and fields of research is unclear.

Implementation research is the study of methods to promote the uptake of research findings into routine clinical practice (encouraging implementation of effective clinical interventions and de-implementation of relatively ineffective clinical interventions) and therefore improve the quality and safety of healthcare. It often involves developing and testing approaches to change the behaviour of health professionals and organisations.^9^ To this end it comprises a distinct field within quality and safety research. Traditionally, the aim of PPI has been to involve end users in the research process. Implementation research differs from clinical research in that it generally aims to understand and change health professional behaviour as opposed to patient behaviours. The targeted research users are therefore usually, if not exclusively, health professionals. For example, audit and feedback is widely used as a strategy to improve professional practice against explicit standards by providing health professionals with a summary of their clinical performance over a specified period of time.^10^ Feedback which is unacceptable to, or not acted on by professionals is unlikely to change professional behaviour, and hence fail to improve patient care and outcomes.

However, there is a lack of guidance on the roles of PPI in implementation research. There may be a risk of tokenism if patients and members of the public are expected to provide input on aspects of implementation research with which they have little direct experience (eg, understanding how health professionals might respond to an audit and feedback report). The potential problems with regard to PPI in implementation research are similar to challenges of involving patients in quality improvement initiatives, which often target both patients and clinicians. Armstrong *et al* 11 explored the extent to which PPI could provide meaningful input in a series of three case studies across diverse quality improvement initiatives. While they found that PPI was uniformly useful for patient-facing elements of redesign (eg, advising on and contributing to information materials for patients), it was less apparent how PPI could best be harnessed in initiatives involving more technical systems redesign. Having funding applications within implementation research contingent on having adequate or even ‘strong’ PPI without clarity on how PPI can best be used can cause tensions for researchers who may be tempted to exaggerate the scope of PPI in grant applications in order to obtain funding. Subsequent PPI may not be optimised for their research area or target group, with resultant risks of superficial engagement and inefficient use of limited resources. There is a pressing need to clarify the role of PPI in implementation research to best use the time and effort of both PPI panel members and researchers.

We aimed to generate and define potential roles of PPI in clinical and implementation research respectively, and thereby promote productive and authentic involvement of patient and public representatives in implementation research.

## Methods

Our study was embedded within an existing NIHR-funded programme of implementation research, ’Action to Support Practices Implementing Research Evidence' (ASPIRE).^12^ The ASPIRE PPI Panel comprises nine people (PB, AC, SH, GI, GP, MR, KS, LW, FY) from diverse ethnic, occupational and social backgrounds and considerable collective lay experience in commissioning and governance of healthcare, national clinical audits, patient advocacy, and National Health Service leaders’ management development and community development. Specifically, KS had been the lay panel assessor for a national confidential enquiry, GP and LW were current or former members of clinical commissioning group boards, LW provided input to the Nye Bevan Senior Management Development programme in addition to the PPI input for other research projects, and MR was an elected governor of a hospital trust, and FY, PB, GI, SH and AC were experienced in community development and leadership, as well as patient advocacy. The panel had been contributing to ASPIRE for 4 years prior to this study and thus possessed a good working knowledge of implementation research. For most panel members the ASPIRE programme represented their first experience of providing representation on an implementation research project. The PPI panel contributed to the design, conduct and interpretation of findings for this consensus study.

We used a modified RAND consensus process,^13^ comprising four face-to-face meetings and completion of two online surveys. We chose the RAND process over others (eg, Delphi method) as it allowed panellists to make both private decisions using structured questionnaires before meeting to discuss decisions and disagreements face-to-face, and provided an explicit method of aggregating judgements (see Murphy *et al.*,^13^ for more details on different consensus methods). Our 11-member consensus panel comprised all nine members of the ASPIRE PPI Panel, and two ASPIRE researchers experienced in both implementation and clinical research (TW, RRCM). We deliberately weighted the panel towards PPI members to ensure that their reflections and experience would form the majority contribution. We had weighted an earlier panel addressing clinical behaviour towards professionals to ensure that we sufficiently accounted for their insights and perspectives.^14^ We felt it was appropriate to reverse the weighting for this panel given the focus on PPI roles in research.

We drew on available literature and panel discussion in the first face-to-face meeting to identify potential PPI roles and challenges in clinical and implementation research. An initial list of roles was compiled by KG-B, and refined following input from the PPI panel and researchers (RF, RRCM and TW) during the first face-to-face meeting. Based on this we developed a 36-item online questionnaire containing 21 statements related to the roles of PPI and 15 statements related to the challenges of PPI in research. We used five core themes for roles: priority setting and shaping research questions (identifying what the problem is, what needs are most important to address and specific questions to be answered by the research); planning research (influencing design of research before and/or after funding); conducting research (guiding how the research will be carried out to ensure suitability for participants); interpreting findings (learning about the findings of the research and expressing opinions on what findings might mean); and sharing and using research knowledge (sharing knowledge gained with others either verbally or in writing and using that knowledge to guide future research).

All items were rated using a 9-point Likert scale, with most requiring indication of the level of agreement (1=strongly disagree, 9=strongly agree). For items describing challenges of PPI, the scaling depended on the item (eg, ‘PPI is challenging because expectations are 1=clear, 9=unclear). We piloted the survey with five individuals without academic roles but an understanding of the ASPIRE programme, and refined it to ensure comprehension and usability.

We invited two ‘expert witnesses’ (researchers highly experienced in PPI, and the involvement of wider stakeholders in research) to the second face-to-face meeting; they presented their experience and opinions around the use of PPI in clinical and implementation research. We aimed to provide varying examples of other types of research which the panel could use to triangulate their own experiences within the ASPIRE programme. For the purpose of the consensus process, clinical research was defined as the following:

Studies using human participants interested in outcomes related to health (ie, improvement in health condition). For example, this might be a study testing the effects of a new inhaler in people with asthma. This evaluates the impact an intervention has on patients.

Implementation research was defined as the following:

The study of methods to promote the uptake of research findings into routine clinical practice. This aims to improve the quality and safety of health care by testing approaches to change the behaviour of health professionals and organisations. For example, this might be the type of work we’ve been doing in ASPIRE, including delivering practice performance data or providing educational meetings to practice staff. This looks at how interventions are implemented.

One week after the expert witness face-to-face meeting, the panel were asked to independently rate each role and challenge statement in an online survey separately for both clinical and implementation research. Participants responded to each item twice, that is, separately to reflect their views regarding clinical and implementation research, completing 72 ratings in total. Panellists were able to record thoughts or comments in free-text fields. High disagreement was defined where at least three panellists scored an item at the lower end of the scale (1–3) and at least three scored it at the higher end of the scale (7–9). Moderate disagreement was defined as at least two panellists scoring at either end of the scale. High or moderate disagreement indicated a lack of consensus. The absence of high or moderate disagreement indicated consensus. Median ratings were calculated for each statement. These ratings were fed back to panellists along with free-text comments at the third meeting. In this meeting disagreements were discussed by the panel, with a view to promoting, but not forcing, consensus.

Following the third meeting, all panellists independently rerated all items online. Median scores and areas of disagreement were recalculated. Statements related to PPI roles were split into those with high support (eg, a median score of 7–9, towards the ‘strongly agree’ anchor of the scale) and consensus (ie, the items failed to meet our criteria for moderate/high disagreement); those with weaker support (eg, a median score of 4–6, within the midpoint of the scale) and consensus; those with no support (eg, a median score of 1–3, towards the ‘strongly disagree’ anchor of the scale) and consensus; and items with no consensus. The fourth panel meeting discussed findings and agreed recommendations for the role of PPI in implementation research.

## Results

Ten panellists (two researchers and eight PPI members) completed both online surveys. In the first survey, disagreements were apparent for 37.5% of statements (27/72, 17 of these relating to implementation research). Seven panellists (two researchers and five PPI members) met to discuss areas of disagreement, and notes of the meeting were circulated to all panel members. After the second online survey, the level of disagreement fell to 22.2% of statements (16/72, 10 of these related to implementation research; online supplementary file 1). Median scores for each statement for both clinical and implementation research after the second online rating can be found in online supplementary file 2.

10.1136/bmjqs-2017-006954.supp1 Supplementary file 1

10.1136/bmjqs-2017-006954.supp2 Supplementary file 2

### Roles of PPI

After administration of the second online survey, for clinical research, there was strong support and consensus for the role of PPI across all elements on the research project (20/21 roles). There was weaker support and consensus for the role of advising on intervention sustainability after research completion. There were no areas of disagreement concerning the role of PPI in clinical research.

For implementation research, panellists agreed on and strongly supported eight roles (within the categories of priority setting and shaping research questions, planning research, and sharing and using research knowledge), agreed on and weakly supported seven roles (within planning research, conducting research, interpreting findings, and sharing and using research knowledge), and did not achieve any consensus on six roles (within planning research, conducting research, and sharing and using research knowledge).

In the final meeting, the panel suggested amendments to further clarify roles of PPI in implementation research and agreed recommendations (boxes 1–3) and online supplementary file 3. The panel agreed that roles with strong support and consensus were areas where PPI had a clear and valuable contribution to make in regard to implementation research. Roles with consensus but weaker support could potentially contribute towards PPI in research; however, further work was needed to explicate their value. The remaining and more contested roles were acknowledged. We propose that researchers can confidently focus on engaging PPI in roles outlined in box 1, and to a lesser extent box 2, within implementation research. Researchers engaging PPI in roles with weaker support (outlined in box 2) and the contested roles (outlined in box 3) should ensure they have appropriate strategies in place to evaluate the contribution of PPI within this context in order to further share learning about the potential of PPI for these roles. For example, researchers could record the perceived success of PPI representative and researcher contributions, as well as recording time, effort and costs associated with involvement.

Box 1 **Roles for patient and public involvement (PPI) in implementation research with strong support**

Priority setting/shaping research questions:

- Setting priority areas for research (eg, what health conditions or outcomes to study).
- Helping to shape research questions and identifying specific research questions.

Planning research:

- Advising on acceptable methods of obtaining consent from research participants (eg, health professionals).
- Reviewing and commenting on applications for research funding.

Conducting research:

- Agenda setting for PPI meetings in collaboration with the research team.
- Providing a governance function to ensure that researchers are acting responsibly (note: this is contingent on PPI members being given the right information by the research team at the appropriate time).

Sharing and using research knowledge:

- Sharing knowledge and learning about the research to other relevant stakeholders (eg, by giving verbal updates, writing reports, presenting at or attending conferences and meetings).
- Guiding the direction of future research.

Box 2 **Roles for patient and public involvement in implementation research with weaker support**

Planning research:

- Advising on potential methods of recruiting research participants (eg, health professionals).
- Conducting research: this role can involve helping to guide how the research will be carried out to ensure it is suitable for participants.
- Helping to inform the content of research materials (eg, information sheets, questionnaires) targeted at health professionals.
- Pretesting research materials and methods (eg, reading and amending information sheets and questionnaires to ensure that they are suitable for research participants).
- Providing a governance function to protect the rights, independence and freedom of choice of research participants (eg, health professionals).

Interpreting findings:

- Reviewing findings to see how the research is progressing (eg, interim analyses within randomised controlled trials).

Sharing and using research knowledge:

- Providing unique knowledge through having personal experience of health conditions or through working closely with target participants.
- Talking to others on the researchers’ behalf or signposting to appropriate groups to meet with to discuss the research.

Box 3 **Contested roles of patient and public involvement in implementation research**

Planning research:

- Advising on the acceptability of the study design and methods for the research participants (eg, health professionals).*

Conducting research:

- Guiding discussions about intervention content to try and change behaviours of the target participants (eg, health professionals).*
- Ensuring that developed interventions are feasible and could be successfully delivered to target participants (eg, health professionals).*
- Ensuring that developed interventions are acceptable to target participants (eg, health professionals).†
- Advising on the likely sustainability of the intervention after the study has ended.*

Sharing and using research knowledge:

- Providing personal insight into how interventions may be received by the target participants (eg, health professionals).*

*Moderate disagreement.†High disagreement.

10.1136/bmjqs-2017-006954.supp3 Supplementary file 3

### Challenges of PPI

Challenges were generally rated around the midpoint of each scale, reflecting some level of uncertainty or ambivalence (eg, panellists neither strongly agreed nor strongly disagreed with the items, see online supplementary file 2). Subsequent panel discussions indicated that many were context-specific and thus difficult to rate. However, the panel agreed that PPI was a good use of time and disagreed with the statement that PPI members lack sufficient knowledge and understanding to guide research appropriately. There were differences in ratings of challenges for the different types of research. Panellists judged the risk of PPI being tokenistic (eg, box-ticking to please funders) and difficulties in engaging the public as higher for implementation research than for clinical research, while clarity of expectations for PPI was lower. However, the panel considered that the risk of PPI being used purely to gain consent and legitimacy for research was higher for clinical research.

## Discussion

We have proposed a set of roles to promote productive and authentic PPI in implementation research. Although PPI in health research is receiving increasing attention,^2–5 7 8 15–17^ our work is novel in explicitly exploring PPI in relation to implementation research, where health professionals rather than patients are often the main focus of research.

We used a structured consensus method, involving experienced PPI members and implementation researchers. Agreement on the role of PPI varied across clinical and implementation research fields. While we found strong support for the value of PPI across all elements of the research process for clinical research, support for the role of PPI in implementation research varied. There was less consensus on elements of the research process which pertained to the conduct of research with health professionals (eg, discussing acceptability, content and feasibility of interventions, providing personal insights into how interventions might be received, and advising on acceptability of study methods). Our panel judged that PPI members could provide general advice on the development of research methods and interventions for implementation research projects. Furthermore, PPI members can play key advocacy roles in, for example, expressing support for recruitment methods to promote health professional participation in research,^12^ in a similar way to which they can act as a ‘technology of persuasion’ in quality improvement.^11^ Nevertheless, the panel recognised that input from the targeted participants (eg, health professionals) is vital as they are the ‘users’ of any developed intervention. It is perhaps a self-evident reminder that the ‘professional’ voice is also valued in the development, conduct and management of implementation research.

There is broad recognition of the importance of defining and involving key users of research, be they patients, professionals or policy-makers, early in and throughout the research process to enhance related knowledge transfer.^18^ We acknowledge that implementation research and interventions can also focus on patients as well as professionals, for example, in considering decision aids or interventions to promote medication adherence.^19^ Indeed, targeting patients as well as professionals can sometimes enhance the effects of implementation interventions.^20^ Therefore, user involvement needs to be tailored according to the scope of any given implementation problem and project. For implementation research interventions that target behaviours of health professionals and patients, it is important that both of these groups are involved in the research process. For this to work effectively it needs to be recognised that the input of these diverse stakeholder groups might be best used at different stages of the research process. Moreover, bringing these two groups together will likely bring fresh challenges for the effective facilitation of PPI activities. More research needs to be done to explore how best to bring both patients’ and professionals’ perspectives together to inform implementation research projects.

While our panel generally supported PPI across different phases of the research process, we also acknowledge that PPI may also have unintended or negative impacts. Within the current consensus study, we identified tokenistic PPI as a particular risk within implementation research. This finding is consistent with recent systematic reviews on the impact of PPI in research which also identified related challenges such as researchers not understanding the contribution of PPI to research.^4 7^ Within limited project budgets and timescales, researchers need to make trade-offs in considering how and to what extent to involve each type of user group, and negotiate these potential roles with PPI representatives. We hope that our recommended roles will be useful for researchers and patient and public partners to jointly plan, review and refine PPI across different aspects and phases of the research process, and ensure the value of PPI is maximised.

The main limitation of our study is that we involved only one PPI panel from one implementation research programme based in primary care. We judged it was important to have a panel experienced in the delivery of implementation research which could draw on their own experiences in considering the contributions of PPI. We do not claim that our panel’s views are representative of those engaged in other implementation research programmes. Similarly, the perspectives and inputs of our research team may not necessarily be representative of those across the wider spectrum of implementation research. Future work should aim to explore views of PPI representatives and researchers about the extent to which our recommended PPI roles are relevant for these different topic areas. Our interactive consensus method encouraged the expression and discussion of contrasting perspectives; we notably did not achieve consensus around a number of roles, but we see this as highlighting problematic issues that need further working through rather than as a failure to achieve (or even force) consensus. We also primarily weighted the panel towards PPI members. Alternative consensus methods, including different weighting of PPI versus research members, may have resulted in different findings. We therefore invite others to build on our work and improve on our methods.

Although PPI is generally reported as having a positive impact on research outputs, there is currently limited scientific evidence of its impact.^4^ Given the consistent policy interest in PPI in research and associated use of resources (not least the time of PPI members), this represents a clear gap. A major problem is that PPI is inconsistently reported across studies,^21^ and can be used by researchers to encompass a range of roles and inputs from simply consultation to genuine involvement and coproduction.^21^ A standardised approach to reporting is required to improve understanding of how PPI adds value to research and underpin future evidence synthesis. The ‘Guidance for Reporting Involvement of Patients and the Public: GRIPP2’ reporting checklist^21^ offers a framework for researchers to ensure that PPI is consistently and accurately reported. We identified 21 potential roles of PPI across both implementation and clinical research (see online supplementary file 3). We suggest these can augment the GRIPP2 checklist by allowing researchers to report more precisely how PPI input is captured during different stages of the research process. We also suggest that they are used to guide both clinical and implementation researchers in planning exactly how they wish to engage PPI members. Future research could also usefully focus on guidance for the reporting and evaluation of the input of health professionals (in addition to patients and the public) to implementation research projects.

## Conclusion

There is strong support for a range of suggested roles for PPI in implementation research. Researchers and their PPI partners need to consider how best to tailor involvement according to the needs of individual research projects. Research funders should be equally aware of the benefits of targeted involvement and that a ‘one size fits all’ approach to PPI risks tokenism and misuse of limited human resources. We share our recommended roles in the hope that they can promote productive and authentic PPI in implementation research, they will inform reporting for future evidence synthesis, and that others will improve on our work.
