# Supplementary material for: Role of patient and public involvement in implementation research: a consensus study
Source: BMJ Qual Saf. 2018 Apr 17;27(10):858–64. doi: 10.1136/bmjqs-2017-006954 (PMC6166593; doi:10.1136/bmjqs-2017-006954)
Supplement: Supplementary file 1 [file bmjqs-2017-006954supp001.doc]

Supplementary File 1: Summary of disagreements from first and second online rating

|  | **First online rating** | | | **Second online rating** | | |  |
| --- | --- | --- | --- | --- | --- | --- | --- |
| **Statement** | **N^a^** | **Clinical^b^** | **Implementation^b^** | **N^a^** | **Clinical^b^** | **Implementation^b^** | **Disagreement reduced?** |
| **Roles of PPI^c^** |  |  |  |  |  |  |  |
| PPI plays an important role in advising on the acceptability of study design (e.g., questionnaire, interview etc.) and methods (e.g., what will study participants be asked to do, how often etc.)  ***(planning research)*** | 9 |  | Moderate | 9 |  | Moderate | No |
| PPI plays an important role in advising on potential methods of recruiting research participants  ***(planning research)*** | 9 |  | Moderate | 9 |  |  | Yes |
| PPI plays an important role in guiding discussions about what interventions should include (i.e., content of the intervention) to try and change behaviours of the target participants.  ***(conducting research)*** | 10 |  | High | 10 |  | Moderate | Partially |
| PPI plays an important role in ensuring the intervention is feasible (i.e., that it could actually be delivered successfully to the target participants)  ***(conducting research)*** | 10 |  | Moderate | 10 |  | Moderate | No |
| PPI plays an important role in ensuring that the intervention is acceptable to the target participants  ***(conducting research)*** | 10 |  | High | 10 |  | High | No |
| PPI plays an important role in advising on the likely sustainability of the intervention after the study has ended (i.e. can it be continued without research support?)  ***(conducting research)*** | 10 |  | Moderate | 10 |  | Moderate | No |
| PPI plays an important role in helping to inform the content of research materials (e.g. information sheets, questionnaires etc.) **^a^**  ***(conducting research)*** | 10 |  | Moderate | 10 |  |  | Yes |
| PPI plays an important role in providing unique knowledge through having personal experience of conditions or through working closely with target participants  ***(sharing and using research knowledge)*** | 10 |  | Moderate | 10 |  |  | Yes |
| PPI plays an important role in providing personal insight into how interventions may be received by the target participants  ***(sharing and using research knowledge)*** | 10 |  | Moderate | 10 |  | Moderate | No |
| PPI plays an important role in talking to others on researchers’ behalf or signposting appropriate groups to meet with to discuss research  ***(sharing and using research knowledge)*** | 10 |  | Moderate | 10 |  |  | Yes |
| **Challenges of PPI** |  |  |  |  |  |  |  |
| Poor decisions may be made if there are conflicting interests between PPI panel members, or between the panel and the researchers (1 strongly disagree – 9 strongly agree) | 10 | High | Moderate | 10 |  |  | Yes |
| Evidence of the value of PPI is (1 plentiful – 9 limited) | 10 | High |  | 10 | Moderate |  | Partially |
| PPI groups are (1 representative – 9 not representative) | 10 | Moderate |  | 10 |  |  | Yes |
| PPI runs the risk of being tokenistic (i.e. box ticking to please funders) (1 strongly disagree -9 strongly agree) | 10 | Moderate |  | 10 |  |  | Yes |
| PPI members find understanding information and its relevance (1 easy – 9 difficult) | 10 |  | High | 9 |  |  | Yes |
| The setting (e.g., an academic institution) and prospect of speaking to someone with a title is (1 not intimidating – 9 intimidating) | 10 | Moderate |  | 10 | Moderate |  | No |
| Representing a large group of people is (1 comfortable – 9 uncomfortable) | 10 | Moderate | Moderate | 10 | Moderate | Moderate | No |
| Engaging the public in research is (1 easy – 9 difficult) | 10 | High |  | 10 | Moderate | Moderate | Partially (Clinical)  / Increased  (Implementation) |
| Meeting research demands (e.g., keeping up to date with work set between meetings, attending meetings when have other commitments) is (1 easy – 9 difficult) | 9 | Moderate | Moderate | 10 | High | High | Increased |
| The inclusion of PPI leads to increased costs in researchers' time and resources (1 strongly disagree – 9 strongly agree) | 9 | Moderate | Moderate | 8 |  |  | Yes |
| PPI members lack sufficient knowledge and understanding of the research process to appropriately direct research (1 strongly disagree – 9 strongly agree) | 10 |  | Moderate | 10 |  |  | Yes |
| The risk that PPI may be used purely to gain consent from target participants and legitimacy for the work is (1 low – 9 high) | 10 | Moderate | Moderate | 10 | High | High | Increased |

Notes ^a^ Missing data accounts for N<10; ^b^ moderate disagreement = at least two panellists scoring at bottom of scale (1-3) and at least two scoring at high end of scale (7-9); high disagreement = at least three scoring at bottom, and at least three scoring at high end; ^c^ response scale for ‘role’ statements range from 1, strongly disagree to 9, strongly agree.
