# Supplementary material for: Role of patient and public involvement in implementation research: a consensus study
Source: BMJ Qual Saf. 2018 Apr 17;27(10):858–64. doi: 10.1136/bmjqs-2017-006954 (PMC6166593; doi:10.1136/bmjqs-2017-006954)
Supplement: Supplementary file 2 [file bmjqs-2017-006954supp002.doc]

Supplementary File 2 Median ratings for roles and challenges of PPI at end of consensus exercise

| **Statement** | **Research area** | **N^a^** | **Median** | **Notes** |
| --- | --- | --- | --- | --- |
| ***ROLES^b^*** |  |  |  |  |
| ***Priority setting / shaping research questions*** |  |  |  |  |
| PPI plays an important role in setting priority areas for research (e.g. what health conditions or outcomes to study) | Clinical | 10 | 8.0 | Stronger support |
|  | Implementation | 10 | 8.0 | Stronger support |
| PPI plays an important role in helping to shape research questions (for example, identifying the specific topic the research will address, e.g., what resources are available to change GP's risky prescribing behaviours?) | Clinical | 10 | 7.5 | Stronger support |
|  | Implementation | 10 | 7.0 | Stronger support |
| ***Planning research*** |  |  |  |  |
| PPI plays an important role in advising on the acceptability of study design (e.g., questionnaire, interview etc) and methods (e.g., what will study participants be asked to do, how often etc) | Clinical | 9 | 9.0 | Stronger support |
|  | Implementation | 9 | 7.0 | No consensus |
| PPI plays an important role in advising on potential methods of recruiting research participants | Clinical | 9 | 9.0 | Stronger support |
|  | Implementation | 9 | 6.0 | Weaker support |
| PPI plays an important role in advising on acceptable methods of obtaining consent from research participants | Clinical | 10 | 9.0 | Stronger support |
|  | Implementation | 10 | 7.0 | Stronger support |
| PPI plays an important role in assisting with grant proposals/applications (for example, by helping to write plain English summaries) | Clinical | 10 | 7.5 | Stronger support |
|  | Implementation | 10 | 7.0 | Stronger support |
| ***Conducting research*** |  |  |  |  |
| PPI plays an important role in guiding discussions about what interventions should include (i.e., content of the intervention) to try and change behaviours of the target participants. | Clinical | 10 | 7.5 | Stronger support |
|  | Implementation | 10 | 6.5 | No consensus |
| PPI plays an important role in ensuring the intervention is feasible (i.e., that it could actually be delivered successfully to the target participants) | Clinical | 10 | 8.0 | Stronger support |
|  | Implementation | 10 | 6.5 | No consensus |
| PPI plays an important role in ensuring that the intervention is acceptable to the target participants | Clinical | 10 | 9.0 | Stronger support |
|  | Implementation | 10 | 5.0 | No consensus |
| PPI plays an important role in advising on the likely sustainability of the intervention after the study has ended (i.e. can it be continued without research support?) | Clinical | 10 | 6.5 | Weaker support |
|  | Implementation | 10 | 5.0 | No consensus |
| PPI plays an important role in helping to inform the content of research materials (e.g. information sheets, questionnaires etc.) | Clinical | 10 | 9.0 | Stronger support |
|  | Implementation | 10 | 6.5 | Weaker support |
| PPI plays an important role in pre-testing research materials and methods (e.g. reading and amending information sheets and questionnaires to ensure that they are suitable for participants) | Clinical | 10 | 9.0 | Stronger support |
|  | Implementation | 10 | 6.5 | Weaker support |
| PPI plays an important role in arranging PPI Panel meetings (for example, meeting content, agenda setting) | Clinical | 10 | 7.0 | Stronger support |
|  | Implementation | 10 | 7.0 | Stronger support |
| PPI plays an important role in making sure researchers act responsibly (e.g. acting in line with the needs of target participants, and ensuring that funds are used appropriately) | Clinical | 9 | 9.0 | Stronger support |
|  | Implementation | 9 | 7.0 | Stronger support |
| PPI plays an important role in protecting the rights, independence and freedom of choice of participants | Clinical | 10 | 8.0 | Stronger support |
|  | Implementation | 10 | 6.0 | Weaker support |
| ***Interpreting findings*** |  |  |  |  |
| PPI plays an important role in reviewing findings to see how the research is progressing (for example, is the intervention working) and what impact this might have for other areas | Clinical | 10 | 8.0 | Stronger support |
|  | Implementation | 10 | 6.5 | Weaker support |
| ***Sharing and using research knowledge*** |  |  |  |  |
| PPI plays an important role in providing unique knowledge through having personal experience of conditions or through working closely with target participants | Clinical | 10 | 8.5 | Stronger support |
|  | Implementation | 10 | 6.0 | Weaker support |
| PPI plays an important role in providing personal insight into how interventions may be received by the target participants | Clinical | 10 | 8.0 | Stronger support |
|  | Implementation | 10 | 5.0 | No consensus |
| PPI plays an important role in talking to others on researchers’ behalf or signposting appropriate groups to meet with to discuss research | Clinical | 10 | 7.5 | Stronger support |
|  | Implementation | 10 | 6.5 | Weaker support |
| PPI plays an important role in sharing knowledge gained from participation (for example, verbally to other relevant groups, writing reports, presenting at or attending conferences) | Clinical | 10 | 7.0 | Stronger support |
|  | Implementation | 10 | 7.0 | Stronger support |
| PPI plays an important role in guiding the direction of future research (for example, by highlighting what is missing from current research) | Clinical | 10 | 7.0 | Stronger support |
|  | Implementation | 10 | 7.5 | Stronger support |
| ***CHALLENGES*** |  |  |  |  |
| Poor decisions may be made if there are conflicting interests between PPI panel members, or between the panel and the researchers (1 strongly disagree -9 strongly agree) | Clinical | 10 | 4.5 |  |
|  | Implementation | 10 | 5.0 |  |
| Evidence of the value of PPI is (1 plentiful – 9 limited) | Clinical | 10 | 6.0 | No consensus |
|  | Implementation | 10 | 6.0 |  |
| PPI groups are (1 representative – 9 not representative) | Clinical | 10 | 4.5 |  |
|  | Implementation | 10 | 5.5 |  |
| PPI runs the risk of being tokenistic (i.e. box ticking to please funders) (1 strongly disagree – 9 strongly agree) | Clinical | 10 | 6.5 |  |
|  | Implementation | 10 | 8.0 |  |
| PPI members find understanding information and its relevance (1 easy – 9 difficult) | Clinical | 9 | 5.0 |  |
|  | Implementation | 9 | 6.0 |  |
| PPI members can clearly see what their involvement has specifically achieved (at any stage of the research process)(1 strongly disagree – 9 strongly agree) | Clinical | 10 | 5.0 |  |
|  | Implementation | 10 | 5.5 |  |
| PPI is challenging because expectations are (1 clear - 9 unclear) | Clinical | 10 | 4.0 |  |
|  | Implementation | 10 | 5.5 |  |
| The setting (e.g., an academic institution) and prospect of speaking to someone with a title is (1 not intimidating – 9 intimidating) | Clinical | 10 | 5.0 | No consensus |
|  | Implementation | 10 | 5.0 |  |
| Representing a large group of people is (1 comfortable – 9 uncomfortable) | Clinical | 10 | 5.0 | No consensus |
|  | Implementation | 10 | 5.0 | No consensus |
| PPI feels like a (1 good use of time - 9 waste of time) | Clinical | 9 | 1.0 |  |
|  | Implementation | 10 | 2.0 |  |
| Engaging the public in research is (1 easy – 9 difficult) | Clinical | 10 | 5.0 | No consensus |
|  | Implementation | 10 | 6.5 | No consensus |
| Meeting research demands (e.g., keeping up to date with work set between meetings, attending meetings when have other commitments) is (1 easy – 9 difficult) | Clinical | 10 | 5.0 | No consensus |
|  | Implementation | 10 | 5.5 | No consensus |
| The inclusion of PPI leads to increased costs in researchers' time and resources (1 strongly disagree – 9 strongly agree) | Clinical | 8 | 5.5 |  |
|  | Implementation | 8 | 5.5 |  |
| PPI members lack sufficient knowledge and understanding of the research process to appropriately direct research (1 strongly disagree – 9 strongly agree) | Clinical | 10 | 3.0 |  |
|  | Implementation | 10 | 3.0 |  |
| The risk that PPI may be used purely to gain consent from target participants and legitimacy for the work is (1 low - 9 high) | Clinical | 10 | 5.0 | No consensus |
|  | Implementation | 10 | 3.5 | No consensus |

Notes: ^a^ Missing data accounts for N<10; ^b^ response scale for ‘role’ statements range from 1, strongly disagree to 9, strongly agree.
