# Supplementary material for: Role of patient and public involvement in implementation research: a consensus study
Source: BMJ Qual Saf. 2018 Apr 17;27(10):858–64. doi: 10.1136/bmjqs-2017-006954 (PMC6166593; doi:10.1136/bmjqs-2017-006954)
Supplement: Supplementary file 3 [file bmjqs-2017-006954supp003.doc]

**Supplementary File 3: Framework of roles for Patient and Public engagement with research**

| **Research Stage** | **Role** | **Clinical Research** | **Implementation Research** |
| --- | --- | --- | --- |
| ***Priority setting / shaping research questions:*** *This role can involve identifying what the problem is, what needs are most important to address, and setting out specific questions to be answered by the research* | 1. Setting priority areas for research (e.g. what health conditions or outcomes to study) | **++** | **++** |
|  | 1. Helping to shape research questions and identifying specific research questions | **++** | **++** |
| ***Planning research:*** *this role can involve influencing research both in preparation for applying for funding, and within an already funded project* | 1. Advising on the acceptability of the study design and methods for the research participants (e.g. patients, health professionals) | **++** | **+** |
|  | 1. Advising on potential methods of recruiting research participants (e.g. patients, health professionals) | **++** | **+** |
|  | 1. Advising on acceptable methods of obtaining consent from research participants (e.g. health professionals) | **++** | **++** |
|  | 1. Reviewing and commenting on applications for research funding | **++** | **++** |
| ***Conducting research:*** *This role can involve helping to guide how the research will be carried out to ensure it is suitable for participants* | 1. Guiding discussions about intervention content to try and change behaviours of the target participants (e.g. patients, health professionals) | **++** | **-** |
|  | 1. Ensuring that developed interventions are feasible and could be successfully delivered to target participants (e.g. patients, health professionals) | **++** | **-** |
|  | 1. Ensuring that developed interventions are acceptable to target participants (e.g. patients, health professionals)* | **++** | **-** |
|  | 1. Advising on the likely sustainability of the intervention after the study has ended | **+** | **-** |
|  | 1. Helping to inform the content of research materials (e.g. information sheets, questionnaires) targeted at health professionals | **++** | **+** |
|  | 1. Pre-testing research materials and methods (e.g. reading and amending information sheets and questionnaires to ensure that they are suitable for research participants) | **++** | **+** |
|  | 1. Agenda setting for PPI meetings in collaboration with the research team | **++** | **++** |
|  | 1. Providing a governance function to ensure that researchers are acting responsibly (Note: this is contingent on PPI members being given the right information by the research team at the appropriate time) | **++** | **++** |
|  | 1. Providing a governance function to protect the rights, independence and freedom of choice of research participants (e.g. patients, health professionals) | **++** | **+** |
| ***Interpreting findings:*** *This role can involve learning about the findings of research and expressing your opinion on what the findings might mean and/or if they are useful for other areas of research* | 1. Reviewing findings to see how the research is progressing (for example, interim analyses within randomised controlled trials) | **++** | **+** |
| ***Sharing and using research knowledge:*** *this role can involve sharing the knowledge gained from research with others either verbally or in writing and using that knowledge to guide research.* | 1. Providing unique knowledge through having personal experience of health conditions or through working closely with target participants | **++** | **+** |
|  | 1. Providing personal insight into how interventions may be received by the target participants (e.g. patients, health professionals) | **++** | **-** |
|  | 1. Talking to others on the researchers’ behalf or signposting to appropriate groups to meet with to discuss the research | **++** | **+** |
|  | 1. Sharing knowledge and learning about the research to other relevant stakeholders (for example, by giving verbal updates, writing reports, presenting at or attending conferences and meetings) | **++** | **++** |
|  | 1. Guiding the direction of future research | **++** | **++** |

**NOTE:** ++ Strongly Recommended role, + Recommended role, - role where further evidence of value warranted. Recommendations were based on a consensus panel exercise and should be interpreted with caution. We encourage all researchers to gather evidence of the acceptability, impact and costs associated with PPI to stimulate critical debate and reflection.
